# Supplementary material for: What do malaria program officers want to learn? A survey of perspectives on a proposed malaria short course in Nigeria
Source: PLoS One. 2021 Sep 29;16(9):e0257890. doi: 10.1371/journal.pone.0257890 (PMC8480891; doi:10.1371/journal.pone.0257890)
Supplement: S1 Table — (DOCX) [file pone.0257890.s001.docx]

**S1 Table: Malaria Short Course Thematic Domains and Topics**

| **Basic Malariology** | **Surveillance/data management** |
| --- | --- |
| Parasite phases of development | Sources of data, data generation |
| Malaria Transmission | Health Information system |
| Continuum of malaria control | Data analysis and interpretation |
| **Clinical presentation of Malaria** | Data Utilization |
| Symptoms of malaria | Use of dashboard |
| Signs of malaria | **Use of computers** |
| Myths in recognition of malaria | Software |
| **Malaria Diagnosis** | Report writing |
| Rapid diagnostic test use | Data entry and analysis |
| Microscopy for malaria parasite | **Leadership skills** |
| Malaria diagnostic test results | Mentoring, supervision, accountability |
| **Malaria Treatment** | **Program Management** |
| Knowledge on treatment guideline | Planning activities and using resources |
| Treatment of malaria | Logistics and commodity distribution |
| **Malaria prevention** | Sustainability of malaria control activities |
| Vector Control | **Basic statistics** |
| Long-lasting insecticidal nets | Descriptive statistics |
| Indoor residual spraying | Use of charts, graphs and tables |
| Chemoprophylaxis | **Communication** |
| Seasonal malaria chemoprevention | Communication for public engagement |
| Intermittent preventive treatment for pregnant women | Written communication |
| **Ethics** | Advocacy |
| Introduction to ethics | Use of new technologies |
| Confidentiality | Teaching methods |
| Conflicts of interests |  |

**Bold font represents** **domain**
