# Supplementary material for: What do malaria program officers want to learn? A survey of perspectives on a proposed malaria short course in Nigeria
Source: PLoS One. 2021 Sep 29;16(9):e0257890. doi: 10.1371/journal.pone.0257890 (PMC8480891; doi:10.1371/journal.pone.0257890)
Supplement: S2 Table — (DOCX) [file pone.0257890.s002.docx]

**S2 Table:** Frequency distribution of respondents’ priority rating of topics in selected malaria thematic domains

| **Malaria thematic domains** | **Low**  **n (%)** | **Medium**  **n (%)** | **High**  **n (%)** | **Mean score ±SD** |
| --- | --- | --- | --- | --- |
|  |  |  |  |  |
| **Basic Malariology** |  |  |  |  |
| Parasite phases of development | 44 (11.5) | 68 (17.7) | 272 (70.8) | 2.59±0.7 |
| Malaria Transmission | 23 (6.0) | 84 (21.9) | 277 (72.1) | 2.66±0.6 |
| Continuum of malaria control | 30 (7.8) | 69 (18.0) | 285 (74.2) | 2.66±0.6 |
| **Clinical presentation of Malaria** |  |  |  |  |
| Symptoms of malaria | 33 (8.6) | 93 (24.2) | 258 (67.2) | 2.59±0.6 |
| Signs of malaria | 39 (10.2) | 100 (26.0) | 245 (63.8) | 2.54±0.7 |
| Myths in recognition of malaria | 60 (15.6) | 108 (28.1) | 216 (56.3) | 2.41±0.7 |
| **Malaria Diagnosis** |  |  |  |  |
| Rapid diagnostic test use | 41 (10.7) | 78 (20.3) | 265 (69.0) | 2.58±0.7 |
| Microscopy for malaria parasite | 60 (15.6) | 96 (25.0) | 228 (59.4) | 2.43±0.7 |
| Malaria diagnostic test results | 50 (13.0) | 58 (15.1) | 276 (71.9) | 2.59±0.7 |
| **Malaria Treatment** |  |  |  |  |
| Knowledge on treatment guideline | 26 (6.8) | 58 (15.1) | 300 (78.1) | 2.71±0.6 |
| Treatment of malaria | 34 (8.9) | 55 (14.3) | 295 (76.8) | 2.70±0.6 |
| **Malaria prevention** |  |  |  |  |
| Vector Control | 23 (6.0) | 71 (18.5) | 290 (75.5) | 2.70±0.6 |
| Long-lasting insecticidal nets | 43 (11.2) | 84 (21.9) | 257 (66.9) | 2.56±0.7 |
| Indoor residual spraying | 70 (18.2) | 134 (34.9) | 180 (46.9) | 2.29±0.8 |
| Chemoprophylaxis | 44 (11.5) | 91 (23.7) | 249 (64.8) | 2.53±0.7 |
| Seasonal malaria chemoprevention | 49 (12.8) | 118 (30.7) | 217 (56.5) | 2.44±0.7 |
| IPT* for pregnant women | 27 (7.0) | 83 (21.6) | 274 (71.4) | 2.64±0.6 |
| **Surveillance/data management** |  |  |  |  |
| Sources of data, data generation | 25 (6.5) | 53 (13.8) | 306 (79.7) | 2.73±0.6 |
| Health Information system | 34 (8.9) | 74 (19.3) | 276 (71.9) | 2.63±0.6 |
| Data analysis and interpretation | 25 (6.5) | 55 (14.3) | 304 (79.2) | 2.73±0.6 |
| Data Utilization | 21 (5.5) | 65 (16.9) | 298 (77.6) | 2.72±0.6 |
| Use of dashboard | 44 (11.5) | 64 (16.7) | 276 (71.9) | 2.55±0.7 |
| **Use of computers** |  |  |  |  |
| Software | 44 (11.5) | 64 (16.7) | 276 (71.9) | 2.60±0.7 |
| Report writing | 23 (6.0) | 95 (24.7) | 266 (69.3) | 2.63±0.6 |
| Data entry and analysis | 23 (6.0) | 64 (16.7) | 297 (77.3) | 2.71±0.6 |
| **Leadership skills** |  |  |  |  |
| Mentoring, supervision, accountability | 23 (6.0) | 57 (14.8) | 304 (79.2) | 2.73±0.6 |
| **Program Management** |  |  |  |  |
| Planning activities and using resources | 33 (8.6) | 67 (17.5) | 284 (74.0) | 2.65±0.6 |
| Logistics and commodity distribution | 32 (8.3) | 68 (17.7) | 284 (74.0) | 2.66±0.6 |
| Sustainability of malaria control activities | 28 (7.3) | 75 (19.5) | 281 (73.2) | 2.66±0.6 |
| **Basic statistics** |  |  |  |  |
| Descriptive statistics | 43 (11.2) | 96 (25.0) | 245 (63.8) | 2.53±0.7 |
| Use of charts, graphs and tables | 30 (7.8) | 105 (27.3) | 249 (64.8) | 2.57±0.6 |
| **Communication** |  |  |  |  |
| Communication for public engagement | 28 (7.3) | 88 (22.9) | 268 (69.8) | 2.63±0.6 |
| Written communication | 37 (9.6) | 102 (26.6) | 245 (63.8) | 2.54±0.7 |
| Advocacy | 35 (9.1) | 90 (23.4) | 259 (67.5) | 2.58±0.7 |
| Use of new technologies | 38 (9.9) | 89 (23.2) | 257 (66.9) | 2.57±0.7 |
| Teaching methods | 26 (6.8) | 98 (25.5) | 260 (67.7) | 2.61±0.6 |
| **Ethics** |  |  |  |  |
| Introduction to ethics | 41 (10.7) | 102 (26.6) | 241 (62.8) | 2.52±0.7 |
| Confidentiality | 46 (12.0) | 88 (22.9) | 250 (65.1) | 2.53±0.7 |
| Conflicts of interests | 55 (14.3) | 108 (28.1) | 221 (57.6) | 2.43±0.7 |

* IPT: Intermittent preventive treatment for pregnant women, SD: standard deviation
