# Supplementary material for: What do malaria program officers want to learn? A survey of perspectives on a proposed malaria short course in Nigeria
Source: PLoS One. 2021 Sep 29;16(9):e0257890. doi: 10.1371/journal.pone.0257890 (PMC8480891; doi:10.1371/journal.pone.0257890)
Supplement: S1 File — (PDF) [file pone.0257890.s003.pdf]

**NIGERIA FIELD EPIDEMIOLOGY AND LABORATORY TRAINING PROGRAMME**

**MALARIA SHORT COURSE**

**NEEDS ASSESSMENT QUESTIONNAIRE [For potential participants]**

Introduction

The Nigeria Field Epidemiology and Laboratory Training Programme is proposing a short course in malaria for disease control programme officers including malaria control programme officers and other stakeholders with a focus on malaria control. The objective of the course is to improve knowledge and build capacity for malaria control and data translation to policy and interventions among program managers. The course comprises a total of 11-12 days in learning workshops, interspersed with 8 weeks on-the-job field work with supervision in their places of work.

In order to adequately address the needs of the trainees/stakeholders for malaria control, we are conducting a needs assessment. Kindly respond to the questions truthfully and suggest whatever you think will be important in a course on malaria like this one.

Participants: malaria programme managers, malaria focal persons, other diseases programme managers, M&E officers, DSNOs, e.t.c.

---

Serial Number _______________ Date:

1. Age in years: __________ 2. Sex: Male [ ] Female [ ]

3. Highest educational qualification: ________________________

4a. Current institution/place of work: ______________________________

4b. State of place of work: ___________________________

5. Designation: ____________________________

6. Which disease control programme are you working in? ____________________

7. Number of years of working in the disease control programme: _______________

If currently working in malaria programme skip to Q 11

8. If you are not working in malaria control programme currently, have you worked in malaria programme in the past?

1. Yes 2. No

9. How many years ago did you work in the malaria control programme? _________________

**10. List malaria-related workshops attended in the last two years:**

| S/N | Title of course/workshop | Training Institution | Date (month/Year | Duration |
|---|---|---|---|---|
| | | | | |
| | | | | |
| | | | | |
| | | | | |
| | | | | |
| | | | | |
| | | | | |
| | | | | |
| | | | | |

**11. Job Description**
**List important tasks to be performed**

| S/n | Actual job description | Current job/tasks you perform | If the current job is not the actual job description why is this so? |
|---|---|---|---|
| 1 | | | |
| 2 | | | |
| 3 | | | |
| 4 | | | |
| 5 | | | |
| 6 | | | |

**12. Training needs in Malaria and its control**

<u>It is important to read the instruction below before answering question Q13.</u>

Please score the domains of malaria topics you think should be in malaria short course as low priority=1, medium=2, high priority=3)

| **Area** | **Domain** | **Low priority= 1**<br><br>**Medium Priority = 2**<br><br>**High priority =3** | **Suggestion on essential topics to be included** |
|---|---|---|---|
| | | **Score** | |
| Basic Malariology | Parasite phases of development | | |
| | Malaria Transmission | | |
| | Continuum of malaria control: prevention- control – elimination and eradication | | |
| Clinical presentation of Malaria | Symptoms of malaria | | |
| | Signs of malaria | | |
| | Myths in recognition of malaria | | |
| Malaria Diagnosis | Rapid diagnostic test use | | |
| | Microscopy for malaria parasite | | |
| | Malaria diagnostic test results | | |
| **Area** | **Domain** | **Low priority= 1**<br><br>**Medium Priority = 2**<br><br>**High priority =3** | **Suggestion on essential topics to be included** |
| | | **Score** | |

| | | | |
|---|---|---|---|
| Malaria Treatment | Knowledge on treatment guideline | | |
| | Treatment of malaria of various degree of severity | | |
| Malaria prevention | Vector Control | | |
| | Bed net/LLIN | | |
| | Indoor residual spraying (IRS) | | |
| | Larviciding | | |
| | Chemoprophylaxis | | |
| | Seasonal malaria chemoprevention (SMC) | | |
| | Intermittent Preventive Therapy (IPT) | | |
| Surveillance/data management | Sources of data, data generation | | |
| | Health Information system | | |
| | Data analysis and interpretation | | |
| | Data Utilisation | | |
| | Use of dashboard for selected malaria-related indicators, (this auto calculates indicator and display the charts) | | |
| Use of computers | Software e.g Microsoft Excel, Word, power point | | |
| | Report writing | | |
| | Data entry and analysis | | |
| **Area** | **Domain** | **Low priority= 1**<br><br>**Medium Priority = 2**<br><br>**High priority =3** | **Suggestion on essential topics to be included** |
| | | **Score** | |

| | | | |
|---|---|---|---|
| Leadership skills | Mentoring, supervision, accountability, coordination, team building and negotiation | | |
| Programme Management | - Planning activities and<br>using resources<br>- Stakeholder mapping and engagement,<br>- Budgeting,<br>- Development of annual malaria operational plans | | |
| | Logistics and commodity distribution | | |
| | Sustainability of malaria control activities | | |
| Basic statistics | Descriptive statistics (mean, mode, median) | | |
| | Use of charts, graphs and tables | | |
| Communication | Communication for public engagement | | |
| | Written Communication | | |
| | Advocacy | | |
| | Use of new Technologies | | |
| | Teaching methods | | |
| | | | |
| **Area** | **Domain** | **Low priority= 1**<br><br>**Medium Priority = 2**<br><br>**High priority =3** | **Suggestion on essential topics to be included** |
| | | **Score** | |
| Ethics | Introduction to ethics | | |

| | | | |
|---|---|---|---|
| | Confidentiality | | |
| . | Conflicts of interests | | |
| | | | |
| | | | |

13. Do you need any further training in relation to your job description? 1. Yes 2. No

14. If yes, what kind of training do you need?-

________________________________________________

15. What are the three biggest knowledge gaps you/your staff have related to successful implementation of malaria programs?

i. ________________________________________________
ii. __________________________________________________
iii. ___________________________________________________

15. How will this course be of help to you?

_________________________________________________

16. Will this course be useful in your career development/promotions?

__________________________

17. Would your employer be willing to release you for the full duration of the course?
i.e five days for training workshops 2 months apart and 2 days a month after the second training workshop

18. Would your employer support you for the 8 week assignment in your work place?
1. Yes 2. No

19. If you think your employer will not support you, tell us why?

______________________________
