# Supplementary material for: What do malaria program officers want to learn? A survey of perspectives on a proposed malaria short course in Nigeria
Source: PLoS One. 2021 Sep 29;16(9):e0257890. doi: 10.1371/journal.pone.0257890 (PMC8480891; doi:10.1371/journal.pone.0257890)
Supplement: Attachment — Submitted filename: Point-by-point response to editorial and reviwers comments.pdf [file pone.0257890.s005.pdf]

Department of Epidemiology and Medical Statistics,

Faculty of Public Health, College of Medicine,

University of Ibadan, Nigeria.

29 June 2021

The Editor,

PLOS|ONE,

Cover Letter: PLOS ONE Decision: Revision required [PONE-D-21-14537] - [EMID:5efe707e491500a6]

Thank you for the review of our paper titled "**What do malaria program officers want to learn? A survey of perspectives on a proposed malaria short course in Nigeria**". The comments are valuable in terms of enriching the quality of the paper.

For instance, two of our references (nos 2 and 8 as contained in the reference list) which are no longer available online have been replaced, respectively. Also, we have updated data availability statement to read as "Data are fully available without restriction. The de-identified data set file is available from the Zenodo database. https://zenodo.org/record/5067680#.YOA2f-gzbIU

We apologize for the mix-up in the financial disclosure. Yes, "This study was supported by Cooperative Agreement Number U2GGH001876 funded by the United States Centers for Disease Control and Prevention through African Field Epidemiology Network to Nigeria Field Epidemiology and Laboratory Training Program. The funders had no role in study design, data collection and analysis, decision to publish, or preparation of the manuscript". On the online submission form, we have unchecked the statement "The authors received no specific funding for this work", and included the names. Patrick Ngugu, Muhammad Shakir Balogun, Olufemi Ajumobi and Adefisoye Adewole as authors who indirectly received salary from the funder.

Please find the responses to the editorial and reviewer's comments below for your information and attention.

Regards!

Yours sincerely,

Rotimi F. Afolabi

Mobile telephone: +234-806-607-5196

Email address: rotimifelix@yahoo.com

Reviewers Notes and Responses

S/N	Comments	Authors' reply and Amendment Made
I	**Editorial comment - Journal Requirements**	
1.	Please ensure that your manuscript meets PLOS ONE's style requirements, including those for file naming. The PLOS ONE style templates can be found at https://journals.plos.org/plosone/s/file?id=wjVg/PLOSOne_formatting_sample_main_body.pdf and https://journals.plos.org/plosone/s/file?id=ba62/PLOSOne_formatting_sample_title_authors_affiliations.pdf	We appreciate these comments. This has been addressed in the revised manuscript by replacing "**Acknowledgement**" with "**Acknowledgments**"<br>Please refer to the<br>• **Acknowledgments section**
2.	Please review your reference list to ensure that it is complete and correct. If you have cited papers that have been retracted, please include the rationale for doing so in the manuscript text, or remove these references and replace them with relevant current references. Any changes to the reference list should be mentioned in the rebuttal letter that accompanies your revised manuscript. If you need to cite a retracted article, indicate the article's retracted status in the References list and also include a citation and full reference for the retraction notice	Reference number 2 (a report) and 8 are no longer available online and have been replaced with more recent references in the revised version of the manuscript.<br>Thank you.
3.	Please improve statistical reporting and refer to p-values as "p<.001" instead of "p=.000" or "p<0.00". Our statistical reporting guidelines are available at https://journals.plos.org/plosone/s/submission-guidelines#loc-statistical-reporting"	This very important comment is appreciated. We have reflected on the PLOS ONE statistical reporting guidelines and applied it as appropriate in the revised version of the manuscript. For instance, we corrected p<0.000 as p<0.001 in the revised version<br>Kindly refer to<br>• **Table 5**
4.	Please include additional information regarding the survey or questionnaire used in the study and ensure that you have provided sufficient details that others could replicate the analyses. For instance, if you developed a questionnaire as part of this study and it is not under a copyright more restrictive than CC-BY, please include a copy, in both the original language and English, as Supporting Information.	Thanks. This has been attached as a pdf supplementary File S3 Questionnaire (PDF).
5.	We note that you have indicated that data from this study are available upon request. PLOS only allows data to be available upon request if there are legal or ethical restrictions on sharing data publicly. For more information on unacceptable	

	data access restrictions, please see http://journals.plos.org/plosone/s/data-availability#loc-unacceptable-data-access-restrictions.<br><br>In your revised cover letter, please address the following prompts:<br>a) If there are ethical or legal restrictions on sharing a de-identified data set, please explain them in detail (e.g., data contain potentially sensitive information, data are owned by a third-party organization, etc.) and who has imposed them (e.g., an ethics committee). Please also provide contact information for a data access committee, ethics committee, or other institutional body to which data requests may be sent.<br>b) If there are no restrictions, please upload the minimal anonymized data set necessary to replicate your study findings as either Supporting Information files or to a stable, public repository and provide us with the relevant URLs, DOIs, or accession numbers. For a list of acceptable repositories, please see http://journals.plos.org/plosone/s/data-availability#loc-recommended-repositories.<br>We will update your Data Availability statement on your behalf to reflect the information you provide.	The data are available without restriction. The analyzed dataset is available from the Zenodo database.<br>Kindly see below with label the weblink for the uploaded Zenodo data file containing de-identified data set.<br>S1 Dataset. Survey data<br>https://zenodo.org/record/5067680#.YOA2f-gzbIU
6.	We note you have included a table to which you do not refer in the text of your manuscript. Please ensure that you refer to Table 4 in your text; if accepted, production will need this reference to link the reader to the Table.	This is appreciated. We have referred to Table 4 in the revised version of the manuscript.<br>Kindly refer to the<br>**Results section, page 10, line 202**
II	**Reviewer #1: Comments**	
1.	The manuscript is technically sound. I however suggest the following	Thank you for this comment and recommendation.
2.	1. that among the keywords line53 the 'program' be written as program officers	This very important observation is highly appreciated. We have effected the correction by replacing “program” with “program officers”<br>Kindly refer to the<br>• **Keywords, page 3, line 53**<br>For the modification in the revised version of the manuscript.
3.	2. Use MSC in the last sentence Line 100 of the Introduction	We have replaced the word “malaria short course” with “MSC” in the revised version of the manuscript<br>Please refer to

		**Introduction, page 5, line 88**<br><br>For the modification in the revised version of the manuscript
4.	3. line 213 correct Table 5 to Table 4	• We thank the reviewer for this observation!<br>We have corrected Table 5 as Table 4 in the revised version of the manuscript.<br>Kindly refer to the<br>**Results section, page 10, line 202**
5.	4. line 300- delete the unclear reason and replace it with- This may be because the females are more involved in home management of malaria and as care givers , they probably would like to get more information on malaria treatment	• This very important comment is appreciated.<br>• We have replaced the statement “The reason remain unclear” with a modified statement “This may be because the females are more involved in community case management of malaria and as caregivers at home, they probably would like to get more information on malaria treatment” in the revised manuscript.<br><br>• Currently, the Nigeria National Malaria Elimination Program uses community case management of malaria. The home is regarded as part of the community in the current Nigeria national guidelines. We prefer to use the term “community case management of malaria”<br><br>• Kindly refer to<br>**Discussion section, page 15, lines 289 – 291**<br><br>in the revised manuscript
6.	5. line 321 Put full stop after Nigeria. This is to achieve malaria elimination, while taking into -----------	• We have included full top (.) after Nigeria and “This is …” in the revised manuscript as pointed out by the reviewer.<br>Please refer to:<br>**Conclusion section, page 16, line 312**
