# Supplementary material for: What do malaria program officers want to learn? A survey of perspectives on a proposed malaria short course in Nigeria
Source: PLoS One. 2021 Sep 29;16(9):e0257890. doi: 10.1371/journal.pone.0257890 (PMC8480891; doi:10.1371/journal.pone.0257890)
Supplement: Attachment — Submitted filename: Point-by-point response to comments_R1.pdf [file pone.0257890.s006.pdf]

Department of Epidemiology and Medical Statistics,

Faculty of Public Health, College of Medicine,

University of Ibadan, Nigeria.

31 August 2021

The Editor,

PLOS|ONE,

Cover Letter: PLOS ONE Decision: Revision required [PONE-D-21-14537R1] - [EMID:02423f107c6c47a8]

We are quite appreciative for all your efforts to ensuring our paper titled "**What do malaria program officers want to learn? A survey of perspectives on a proposed malaria short course in Nigeria**" is of high standard.

Editor, we did upload our response to both the editorial and the reviewer's comments on July 3 2021 using the editorial manager. In particular, the reviewer's comments as listed in the decision letter tagged "PLOS ONE Decision: Revision required [PONE-D-21-14537] - [EMID:5efe707e491500a6]" were all addressed in our responses. However, we observed that reference number 13 was not addressed (alongside references number 2 and 8) as it is no longer online. We sincerely apologize for this mix-up. We have removed reference number 13 in the cleaned revised version of the manuscript. In addition, the changes and corrections effected based on the reviewer's former comments were yellow in the tracked version of the manuscript.

Please find the responses to the editorial and reviewer's former comments below for your information and attention.

Regards!

Yours sincerely,

Rotimi F. Afolabi

Mobile telephone: +234-806-607-5196

Email address: rotimifelix@yahoo.com

Reviewers Notes and Responses

S/N	Comments	Authors' reply and Amendment Made
I	**Editorial comment - Journal Requirements**	
1.	Please review your reference list to ensure that it is complete and correct. If you have cited papers that have been retracted, please include the rationale for doing so in the manuscript text, or remove these references and replace them with relevant current references. Any changes to the reference list should be mentioned in the rebuttal letter that accompanies your revised manuscript. If you need to cite a retracted article, indicate the article's retracted status in the References list and also include a citation and full reference for the retraction notice	This very important comment is appreciated.<br>• Reference number 13 (**Federal Ministry of Health. National Malaria Vector Control Division. Procurement and supply chain management database. Abuja, Nigeria 2017**) is no longer available online. We have therefore replaced reference numbers 12 and 13 with only reference number 12 in the revised version of the manuscript. Please refer to **Discussion section, page 14, line 265**<br>• The reference list has also been modified accordingly by removing reference number 13 from the list. Also, we have edited the list to ensure the listed references are correct and complete. Kindly refer to **Reference, pages 17-18**<br><br>Thank you.
2.	Additional Editor Comments (if provided):<br>• The Author is yet to respond and revise the manuscript as commented by the reviewer<br>• Let the author respond "	Again, thank you for your keen interest in ensuring our manuscript is enriched. This comment is appreciated. However, all the reviewer's former comments as listed in the decision letter tagged "**PLOS ONE Decision: Revision required [PONE-D-21-14537] - [EMID:5efe707e491500a6]"** were all addressed in our response uploaded on July 3,2021. For the sake of clarity, our responses are hereby represented as follows.
II	**Reviewer #1: Comments (as listed in the decision letter tagged "PLOS ONE Decision: Revision required [PONE-D-21-14537] - [EMID:5efe707e491500a6]")**	
1.	The manuscript is technically sound. I however suggest the following	Thank you for this comment and recommendation.
2.	1. that among the keywords line53 the 'program' be written as program officers	This very important observation is highly appreciated. We have effected the correction by replacing "program" with "program officers"<br>Kindly refer to the

		• **Keywords, page 3, line 53**<br>For the modification in the revised version of the manuscript.
3.	2. Use MSC in the last sentence Line 100 of the Introduction	We have replaced the word "malaria short course" with "MSC" in the revised version of the manuscript<br>Please refer to<br>**Introduction, page 4, line 88**<br>For the modification in the revised version of the manuscript
4.	3. line 213 correct Table 5 to Table 4	• We thank the reviewer for this observation!<br>We have corrected Table 5 as Table 4 in the revised version of the manuscript.<br>Kindly refer to the<br>**Results section, page 10, line 202**
5.	4. line 300- delete the unclear reason and replace it with- This may be because the females are more involved in home management of malaria and as care givers , they probably would like to get more information on malaria treatment	• This very important comment is appreciated.<br>• We have replaced the statement "The reason remains unclear" with a modified statement "This may be because the females are more involved in community case management of malaria and as caregivers at home, they probably would like to get more information on malaria treatment" in the revised manuscript.<br>• Currently, the Nigeria National Malaria Elimination Program uses community case management of malaria. The home is regarded as part of the community in the current Nigeria national guidelines. We prefer to use the term "community case management of malaria"<br>• Kindly refer to<br>**Discussion section, page 15, lines 289 – 291**<br>in the revised manuscript
6.	5. line 321 Put full stop after Nigeria. This is to achieve malaria elimination, while taking into -----------	• We have included full top (.) after Nigeria and "This is …" in the revised manuscript as pointed out by the reviewer.<br>Please refer to:<br>**Conclusion section, page 16, line 312**
